# Demographic and Genetic Patterns of Variation among Populations of *Arabidopsis thaliana* from Contrasting Native Environments

**DOI:** 10.1371/journal.pone.0007213

**Published:** 2009-09-29

**Authors:** Alicia Montesinos, Stephen J. Tonsor, Carlos Alonso-Blanco, F. Xavier Picó

**Affiliations:** 1 Departamento de Ecología Integrativa, Estación Biológica de Doñana (EBD), Consejo Superior de Investigaciones Científicas (CSIC), Sevilla, Spain; 2 Department of Biological Sciences, University of Pittsburgh, Pittsburgh, Pennsylvania, United States of America; 3 Departamento de Genética Molecular de Plantas, Centro Nacional de Biotecnología (CNB), Consejo Superior de Investigaciones Científicas (CSIC), Madrid, Spain; University of Chicago, United States of America

## Abstract

**Background:**

Understanding the relationship between environment and genetics requires the integration of knowledge on the demographic behavior of natural populations. However, the demographic performance and genetic composition of *Arabidopsis thaliana* populations in the species' native environments remain largely uncharacterized. This information, in combination with the advances on the study of gene function, will improve our understanding on the genetic mechanisms underlying adaptive evolution in *A. thaliana*.

**Methodology/Principal Findings:**

We report the extent of environmental, demographic, and genetic variation among 10 *A. thaliana* populations from Mediterranean (coastal) and Pyrenean (montane) native environments in northeast Spain. Geographic, climatic, landscape, and soil data were compared. Demographic traits, including the dynamics of the soil seed bank and the attributes of aboveground individuals followed over a complete season, were also analyzed. Genetic data based on genome-wide SNP markers were used to describe genetic diversity, differentiation, and structure. Coastal and montane populations significantly differed in terms of environmental, demographic, and genetic characteristics. Montane populations, at higher altitude and farther from the sea, are exposed to colder winters and prolonged spring moisture compared to coastal populations. Montane populations showed stronger secondary seed dormancy, higher seedling/juvenile mortality in winter, and initiated flowering later than coastal populations. Montane and coastal regions were genetically differentiated, montane populations bearing lower genetic diversity than coastal ones. No significant isolation-by-distance pattern and no shared multilocus genotypes among populations were detected.

**Conclusions/Significance:**

Between-region variation in climatic patterns can account for differences in demographic traits, such as secondary seed dormancy, plant mortality, and recruitment, between coastal and montane *A. thaliana* populations. In addition, differences in plant mortality can partly account for differences in the genetic composition of coastal and montane populations. This study shows how the interplay between variation in environmental, demographic, and genetic parameters may operate in natural *A. thaliana* populations.

## Introduction

Identifying the genetic mechanisms underlying an organism's adaptive evolution in natural environments and populations is one of the most challenging and synthetic disciplines in plant biology [1]–[6]. To achieve this goal, the genes and molecular pathways controlling ecological and evolutionarily important traits, which are those whose variation confers differential adaptive value to distinct environments [7], have to be identified. For that purpose, the annual plant *Arabidopsis thaliana* provides an ideal genetic model [8] given its particular genetic features (e.g.: small genome, genomic sequences and resources, nucleotide variants among natural populations, easy genetic transformation) that allow the rapid identification of gene function [3], [4]. However, to fully understand the significance of genetic variation in functional terms, one must also identify the traits with ecological and evolutionary relevance in natural populations across *A. thaliana*'s environmental range [2], [3]. This is only possible by studying *A. thaliana*'s population biology in its native environments, which is currently being explored through different approaches.

The first approach explores the extent of geographic structure of genetic variation throughout *A. thaliana*'s distribution range [9]–[15]. This large-scale approach allows the identification of genetic groups that are geographically differentiated as the result of historical processes (i.e.: glacial isolation and inter-glacial colonization) and/or adaptive processes to different environments where the species occurs. Importantly, the assessment of genetic structure is required to map the causal genes responsible for observed natural variation in ecologically important complex traits by genome-wide association analysis to reduce spurious correlations between genotype and phenotype [16].

A second approach is more experimental and uses field-collected populations, usually as accessions from stock centers, or recombinant inbred lines derived from crosses among divergent wild genotypes to measure phenotypes under controlled or field experimental conditions [17]–[25]. These experiments determine the functional consequences of variation in life-history traits, the genetic basis of quantitative traits, the extent of the genetic correlations among traits, and the physiological mechanisms underlying plant responses to environmental stresses. This is of the greatest importance to uncover the causes and constraints of adaptation in natural populations.

The third approach can be referred to as the “demographic approach” and it aims to (i) quantify the interplay of variation in environmental conditions, demographic performance, and genetic composition of natural *A. thaliana* populations *in situ*, and (ii) analyze the long-term consequences of that variation for the persistence of local populations. This is important because selection pressures in natural environments seem to be complex and far from constant across space and time [26], [27] and study of the population biology in the sites in which specific adaptations evolved is needed to understand the forces that have shaped life-history traits and functional adaptations. Furthermore, a demographic approach is central to understanding evolution because propagation of genes and traits depends on survival, fertility, and dispersal of individuals that carry them [28]. To date, this demographic approach is not well developed in *A. thaliana* and only a few field studies have focused on the ecology of seed dormancy [29], [30] or the effects of herbivory on population performance [31], [32].

The goal of this work is to develop this demographic approach by studying natural populations of *A. thaliana* from its native range to assess the extent of environmental, demographic and genetic variation. The populations of study occur in two contrasting geographic regions in NE Spain including coastal low-elevation and montane high-elevation sites. The Iberian Peninsula encompasses a high diversity of anthropic and wild native environments at low and high elevations in which *A. thaliana* can be found [15], [33]. This present study jointly analyzes geographic, environmental, demographic and genetic data from 10 natural populations to provide insights into the population biology of the species. Our demographic approach identifies ecological factors and biological processes that may affect demographic attributes and the genetic diversity and differentiation of populations. This is the first step toward building hypotheses about the population dynamics of *A. thaliana* in its natural environments.

## Materials and Methods

### Plant species and populations

*Arabidopsis thaliana* (L.) Heyhn. (Brassicaceae) is a small annual herb. The plant is a cosmopolitan generalist whose native range is considered to be Europe and the Middle Asian mountain system [34]. The species is self-compatible and self-fertile. *Arabidopsis thaliana* can be found in a wide range of anthropic and wild habitats in the Iberian Peninsula, including agricultural fields, banks and track sides, and openings of deciduous and Mediterranean forests and scrublands [15].

**Figure 1:**
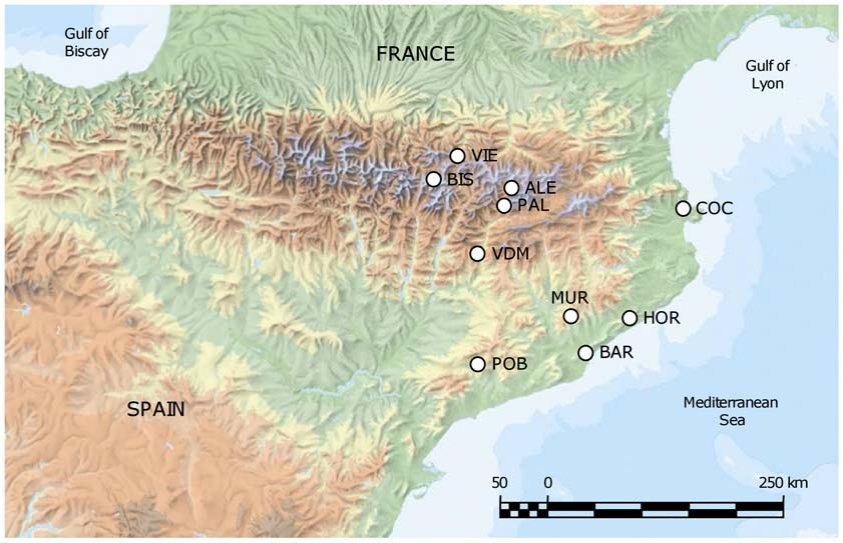
Geographic location of populations. The map shows the location of *A. thaliana* populations of study in coastal (BAR, COC, HOR, MUR, and POB) and montane (ALE, BIS, PAL, VDM, and VIE) areas in NE Spain.

**Figure 2:**
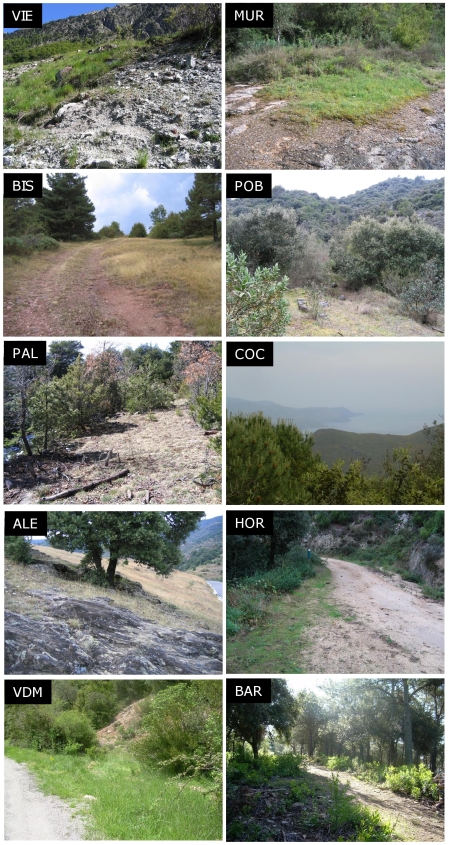
Photographs of study populations. The photographs show the habitat type and the area where permanent plots were laid down. Left and right panels are montane and coastal populations, respectively. Populations are ranked according to their altitude within each region.

We selected 10 *A. thaliana* populations in NE Spain (Figure 1 and Figure 2) that were located in natural areas with some degree of protection (e.g.: inside natural parks). Population codes correspond to three letters of the closest village: ALE, Albet; BIS, Bisaurri; PAL, Pallarols del Cantó; VDM, Vilanova de Meià; VIE, Vielha; BAR, Barcelona; COC, Cabo de Creus; HOR, Hortsavinyà; MUR, Mura; POB, Poblet. Populations were first located in 2004 (POB, MUR, VIE and BIS) and 2006 (COC, BAR, HOR, VDM, ALE and PAL). Distance between population pairs ranged 8–220 km and they were classified into two regions: coastal (along the coastal mountain range of Catalonia) and montane (in the Eastern Pyrenees) populations (Figure 1 and Figure 2). Based on their geographic location, it is well accepted that coastal and montane populations experience Mediterranean and sub-alpine climates, respectively. Such contrasting climates are expected to affect the demographic and genetic performance of *A. thaliana* populations.

### Environmental variables

The latitude, longitude and altitude of each population were recorded with a GPS (Garmin International, Inc., Olathe, KS). The distance from the sea was determined by plotting the shortest straight line from each GPS coordinate to the sea using Google Earth 4.3 (Google Inc., Mountain View, CA). For each population, the following climatic variables were obtained from the Digital Climatic Atlas of Spain (http://opengis.uab.es/wms/iberia/index.htm): mean monthly and mean annual minimum and maximum temperatures, total monthly and total annual precipitation. The landscape in a 78-ha circular area around the GPS coordinates was classified using the CORINE Land Cover Map of Spain (http://www.idee.es) as to the proportion of each of the following cover types: urban, crops/semi-natural grasslands, and primarily woody vegetation. Soil data were obtained by mixing three 5×5 cm soil cores from each population collected in October 2007. Samples were analyzed at the Soil Analysis Service from The Institute for Natural Resources and Agrobiology (IRNAS-CSIC) of Sevilla. The following parameters were estimated: pH, electrical conductivity, organic carbon, organic matter, C/N ratio, proportion of coarse sand, fine sand, slime and clay, and the proportion of N, P, K, Ca and Mg. The fixed effect of region for geographic, climatic, landscape and soil data were analyzed with one-way ANOVAs using population values as replicates within regions.

### Demographic monitoring and data

The COC population was excluded from all demographic surveys for logistic reasons. Three coastal (HOR, MUR and POB) and three montane (ALE, BIS and PAL) populations were used for study of the demographic attributes of the seed bank. The coastal BAR and the montane VIE and VDM were dropped from the seed bank studies for different reasons. The seed bank experimental setup was ruined in BAR by clearing of the understory vegetation and disturbance of the soil in summer 2008. The VIE population grows on a steep river bank with highly unstable soil, mainly rock and gravel, and its movement over winter compromised the experimental setup. We could not collect seed in VDM. In spring 2007, we pooled all available ripe seed from 20–30 plants in each population. A total of 5400 seeds (900 seeds per population ×6 populations) were used for the seed bank experiment. For each population, 100 filled mature seeds were placed in each of nine nylon mesh bags. Sets of three bags (triplets) were buried together at a 5 cm depth. Three groups of triplets were buried with even spacing between them on a 160 cm line 2 m distant from the census transect (described below). Every six months after burial, i.e.: autumn 2007, spring 2008 and autumn 2008, we retrieved one bag per triplet from each population. Retrieved seed bags were kept in a plastic bag at room temperature in the dark for three weeks or less prior to the germination assay. Seeds were classified as filled (round, golden color, inflated) or dead (amorphous, dark color, hollowed) and counted under a Stemi-2000-C stereomicroscope (Carl Zeiss Optical, Inc., Chester, VA). All seeds were then placed in moist Petri dishes at 4°C in darkness for four days and transferred to 22°C in light for four additional days in an AGP-700-ESP incubator (Radiber, S.A., Barcelona, Spain). Seed mortality was calculated as the number of dead seeds divided by the total number of seeds. Seeds previously classified as dead never germinated. Seed germination was calculated as the number of germinated seeds divided by the number of filled seeds.

Typically, a natural *A. thaliana* population is represented by an assemblage of patches differing in the number of plants, area and distance from other patches. In May 2007 we selected one representative patch with flowering *A. thaliana* plants within each population of study so all our demographic data refer to that particular patch within each population. We laid down a 4 m permanent transect through the longest axis containing plants within the patch. At 80 cm distances along the transect groups of three 20×20 cm census plots were established in a line perpendicular to the main transect and separated by 10 cm from each other, totaling 18 plots per transect. The position of each plot was tagged with numbered nails. The overall number of plants recorded among plots in spring 2007 ranged 50–742 plants among populations. During the next growing season, we counted the number of plants per plot and recorded their phenological state (i.e.: vegetative or reproductive) at each of the following five surveys: late October 2007, late December 2007, early March 2008, early April 2008 and mid/late May 2008. In all surveys data from all populations were recorded within one week interval.

We estimated the change in plant density (*ΔN*) between October and December 2007 as *ΔN* = ln(*N_DEC_*)−ln(*N_OCT_*), where *N_DEC_* and *N_OCT_* are the number of plants in December and October, respectively. *ΔN* was estimated in three coastal (BAR, HOR and POB) and four montane (ALE, BIS, PAL and VDM) populations. The MUR and VIE populations were excluded because of nil (VIE) or low (MUR; 10 plants in four plots) number of plants between October and December. Plots with no individuals in either survey were excluded from the analysis (63 of 162 plots). For the rest of plots with at least more than one individual per plot in either survey, we added 1 to all values to avoid problems with 0 values in October or December surveys when taking logarithms. Logarithm values reduced dispersion of data and improved normality. Density values ranged between 0–318 and 0–107 individuals per plot in October and December, respectively. Positive and negative *ΔN* values indicate increase and decrease in plant density between October and December, respectively.

The cotyledons of *A. thaliana* cannot always be distinguished from those of other annual crucifers, while the first true leaves can be distinguished by their color, shape and the presence of stellate trichomes. Therefore the smallest plants recorded during this study had two recognizable cotyledons and the first two leaves (≈0.5 cm rosette diameter). In December 2007 the number of leaves per plant was counted in three coastal (BAR, HOR and POB) and three montane (ALE, PAL and VDM) populations. We excluded MUR, BIS and VIE from the analyses because of nil (MUR and VIE) or low (BIS; five plants) number of plants in December. After December we could not accurately estimate the number of rosette leaves since depredated and senesced leaves could not necessarily be detected.

In May 2007, we selected 1–3 filled fruits from 13–19 plants of different sizes (determined by fruit production) whose total fruit number spanned the observed range in each population (range of plant sizes across populations  = 2–146 fruits per plant). We collected these fruits in four coastal (BAR, HOR, MUR and POB) and four montane (ALE, BIS, PAL and VIE) populations. We could not collect seed in VDM. For each plant, we estimated the mean number of seeds per fruit by counting the number of seeds of each fruit and calculating the mean. Then filled seeds were pooled and weighed to the nearest 0.1 mg using a Sartorious BP61S balance (Sartorius AG, Goettingen, Germany). For each plant, we estimated mean seed weight by dividing total seed weight by total number of seeds. A year later, in mid/late May 2008, when all plants finished their reproduction, we counted the number of aborted flowers, the number of filled fruits, and the number of depredated fruits per individual plant. We estimated these reproductive traits in three coastal (BAR, HOR and POB) and three montane (BIS, VDM and VIE) populations. The HOR population was destroyed in spring 2008 but we estimated reproductive traits for this population from 153 off-transect additional plants. The coastal MUR and montane ALE and PAL populations were excluded from the analyses because of nil (ALE) or low (MUR; three plants, PAL; four plants) number of reproductive plants in spring 2008.

The fixed effect of region (coastal *vs.* montane), the random effect of population nested within regions, and the fixed effect of time (autumn 2007, spring 2008 and autumn 2008) on percent mortality and percent germination of seeds in the soil seed bank was analyzed with Generalized Linear Models. The fixed effects of population and time on the number of plants per population throughout the year were analyzed with a two-way repeated-measures ANOVA in which time was the repeated measure factor with values of October 2007, December 2007, March 2008, April 2008 and May/June 2008. We did not test the region effect on the number of plants per population because of the imbalance of populations between regions for this trait: two coastal (BAR and POB) and five montane (ALE, BIS, PAL, VDM and VIE) populations had enough plots with plants (range 7–18 plots) throughout the year to be included in the analyses. The fixed effect of region and the random effect of population within regions on the change in plant density between October and December were analyzed with a Generalized Linear Model using the total number of plants per plot recorded in October as a covariate to account for density-dependence effects. The fixed effect of region and the random effect of population nested within regions on mean number of rosette leaves in winter, percentage of aborted flowers, total number of filled fruits, and percentage of depredated fruits were analyzed with Generalized Linear Models. The fixed effect of region and the random effect of population nested within regions on mean number of seeds per fruit and mean seed weight were also analyzed with Generalized Linear Models including maternal plant size measured by the total number of fruits as a covariate. When the population within region effect was significant, differences among populations were analyzed with the Student-Newman-Keuls post-hoc test. Because the distribution of individuals among plots was highly skewed, we used pooled individual plants across all plots within populations as replicates rather than means per plot to conduct analyses on rosette size and all reproductive traits. Generally, these parametric tests are quite robust against some deviations from their underlying assumptions (independence, homogeneity of variances, normality and additivity). When necessary, however, variables were transformed for normality, homoscedasticity and linearity, using arcsine transformation for proportions and log transformation for all other values. Statistical analyses were performed using SPSS v.13 statistical software (SPSS, Inc., Chicago, IL).

### DNA isolation, marker genotyping, and genetic data

We collected seed from 20–25 randomly-chosen individuals from the 10 populations of study. Seeds were collected in June 2006 for VDM and in May/June 2007 for all others. Seeds were stored in plastic bags at room temperature in darkness. Three months after collection, seeds were stratified in moist Petri dishes at 4°C in darkness for four days in the incubator. For each individual, 20–40 seeds were sown on trays with standard soil mixture in an air-conditioned greenhouse (22°C day, 15°C night, 16 h day length, and constant high moisture) from The Institute for Plant Biochemistry and Photosynthesis (IBVF-CSIC) of Sevilla. Five weeks after sowing, for each field-collected individual a mix of leaf tissue from 6–10 sister plants was used for maternal DNA isolation. DNA was isolated using a previously described protocol [35] without mercaptoethanol. In the case of BIS, seeds from some individuals did not germinate and the population was represented by 13 individuals, which might have slightly reduced its estimation of genetic diversity.

A total of 188 individuals from 10 populations were genotyped with two sets of presumed neutral nuclear SNP loci previously described [15]. One set contains 96 Col/C24 markers (polymorphic in Central Europe) and the other 47 IP markers (polymorphic in the Iberian Peninsula). This allows the assessment of the SNP ascertainment bias due to the geographic origin of the accessions used for polymorphism discovery [15]. The SNPlex technique (Applied Biosystems, Foster City, CA) was used for genotyping three mixes of 47/48 loci through the CEGEN Genotyping Service (http://www.cegen.org). Twenty-one Col/C24 and one IP SNPs did not amplify and were not taken into account. All SNPs had 1–21% of missing values (mean±SE  = 6.2±0.46%), except three Col/C24 markers that had 36–43% of missing values and were also excluded from the analyses. Forty of 72 Col/C24 and 36 of 46 IP SNPs segregated among individuals and genetic analyses were based on these 76 polymorphic SNPs. Based on 17 duplicated individuals for the 118 SNPs that worked, we estimated a genotyping error of <0.0005%.

We estimated the following genetic diversity parameters: percentage of polymorphic loci (*PL*), mean number of observed alleles per locus (*n_a_*), mean allelic richness per locus (*R_S_*), mean private allelic richness per locus (*R_P_*), mean gene diversity (*H_S_*), and number of multilocus genotypes (*N_H_*). We used FSTAT v.2.9.3 [36] to estimate *n_a_*, *R_S_* and *H_S_*, and HP-Rare v.1.0 [37] to estimate *R_P_*. We do not report observed heterozygosity because previous comparisons between microsatellites and SNP markers indicated that the SNPlex technique detects some heterozygous loci as missing data [15]. Differences between coastal and montane populations for genetic diversity parameters were analyzed with a one-way ANOVA.

Genetic differentiation among regions (coastal *vs.* montane), among populations within regions, and among individuals within populations was estimated by hierarchical analysis of molecular variance AMOVA [38] using the program ARLEQUIN v.3.1 [39]. *F_ST_* statistics [40] and their significances were calculated from 1000 permutations. The relationship between genetic distance (*D*), calculated as the Slatkin's linearized *F_ST_*, *D*  =  *F_ST_*/(1-*F_ST_*) [41], and Euclidean geographic distance among population pairs was estimated using the Isolation-By-Distance Web Service v.3.15 [42]. Genetic and geographic distances were log transformed prior to analysis and the significance of the Mantel test [43] was calculated with 1000 randomizations.

Data were analyzed for the two sets of SNPs separately. As expected IP SNPs yielded higher genetic diversity estimates than Col/C24 SNPs developed from Central European accessions. However, the results from both sets of SNPs were similar and highly correlated (*r*>0.82; *P*<0.004 for *R_S_* and *H_S_*) indicating that marker ascertainment bias is low and that the patterns of genetic diversity and differentiation between groups of populations (see the *Results* Section) are consistent. For the sake of clarity, we only show results from the joint analysis of all SNP markers.

## Results

### Environmental variability between regions

**Figure 3:**
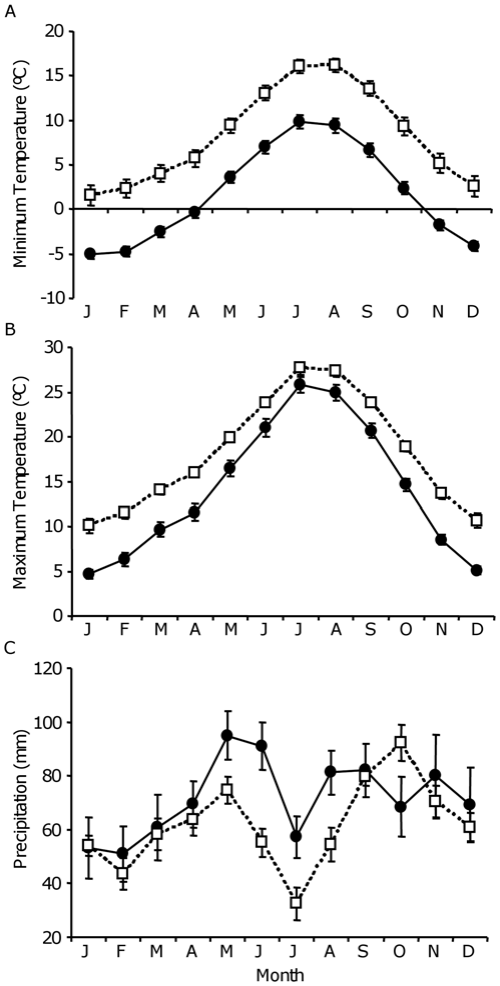
Climatic patterns for coastal and montane regions. The three panels show mean (± SE) values of (A) monthly minimum temperature, (B) monthly maximum temperature, and (C) monthly total precipitation for montane (filled dots) and coastal (hollow cuadrats) populations.

Coastal *A. thaliana* populations were located at lower altitudes and closer to the sea than montane populations (*P*<0.0001 for both parameters; Table 1) and the two regions significantly differed in mean annual minimum and maximum temperatures (*P* = 0.001; Table 1). On average, montane populations had minimum temperatures below 0 between November and April whereas minimum temperatures remained above 0 during the whole year in coastal populations (Figure 3A). Maximum temperatures were higher in coastal than in montane populations throughout the year and peaked in July in both regions (Figure 3B). Annual total precipitation did not differ between groups of populations (*P* = 0.41; Table 1) although the monthly pattern of precipitation differed between regions: the months with the highest precipitation records were October and May for coastal and montane populations, respectively (Figure 3C).

**Table 1:** Environmental parameters of *A. thaliana* populations.

Region	Population	Altitude (m)	Distance from the sea (km)	Minimum annual T (°C)	Maximum annual T (°C)	Accumulated annual rain (mm)	Grasslands and crops (%)	Bushes (%)	Woods (%)
**Mountain**	**ALE**	1225	132	2.2	15.3	606.5	55.2	32.4	12.3
	**BIS**	1450	164	1.1	13.7	1096.2	15.7	36.6	47.8
	**PAL**	1433	127	0.0	13.5	585.9	3.9	57.4	38.7
	**VDM**	975	104	3.2	16.1	716.8	39.9	0.0	60.1
	**VIE**	1620	173	0.5	12.3	1064.7	12.5	48.7	38.9
**Coast**	**BAR**	429	8	10.4	18.0	486.1	0.0	0.0	87.2
	**COC**	519	4	8.9	18.3	719.8	0.0	100.0	0.0
	**HOR**	431	6	8.4	18.6	835.9	0.0	0.0	100.0
	**MUR**	836	33	5.3	16.9	788.0	0.0	42.9	57.1
	**POB**	656	32	7.3	18.6	691.3	0.0	52.6	47.4
**Mountain**		**1340.6 (110.8)**	**140.0 (14.3)**	**1.4 (0.6)**	**14.2 (0.7)**	814.0 (111.1)	**25.4 (9.6)**	35.0 (9.8)	39.5 (7.9)
**Coast**		**574.2 (77.4)**	**16.6 (7.3)**	**8.1 (0.9)**	**18.1 (0.3)**	704.2 (60.1)	**0.0 (0.0)**	39.1 (18.7)	58.3 (17.5)

Average (± SE) values for montane and coastal populations are indicated below. Average values in bold face differ significantly from one another (*P*<0.05).

The differences in landscape variables between groups of populations were only significant for the proportion of crops/semi-natural grasslands (*P* = 0.029; Table 1). Sites of coastal populations were occupied by natural Mediterranean plant communities consisting of mixed forests of oaks, pines and holm oaks alternated with patches of bushy vegetation, whereas montane populations were surrounded by 3.9–55.2% of crops/semi-natural grasslands. The BIS population was the only one with crops whereas the other montane populations were partly occupied by semi-natural grasslands. The BAR population was the only population with urban landscape (12.8%), category that was not included in the analysis. Coastal and montane populations did not significantly differ for soil parameters (all *P*>0.084; data not shown), except for Mg concentration (*P* = 0.043). On average coastal soils (mean±SE  = 308.8±58.1 mg/Kg) had more Mg available than montane soils (141.0±40.3 mg/Kg).

### Demographic traits: seed bank

**Figure 4:**
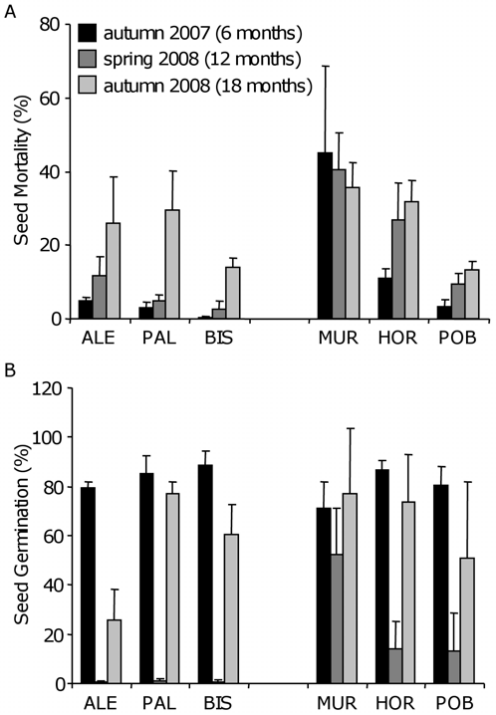
Persistence and germination of seeds buried in the soil seed bank. The two panels show mean (± SE) values of (A) percent seed mortality and (B) percent seed germination of the soil seed bank experiment conducted on montane (ALE, BIS, and PAL) and coastal (HOR, MUR and POB) *A. thaliana* populations. Seeds were collected and buried in local populations in spring 2007 and retrieved periodically every six months: autumn 2007, spring 2008, and autumn 2008.

Mortality of seeds in the soil seed bank did not significantly differ between regions (*P* = 0.20). Population, retrieval time and the interaction between region and retrieval time had a significant effect on seed mortality (*P*<0.037 in all cases). Seed mortality in the soil seed bank clearly increased with time (Figure 4A), ranging from a low of 11.9±4.6% during the first six months to a high of 25.4±3.1% after 1.5 years buried in the soil seed bank. The MUR population was the exception because seed mortality was similarly high in the three surveys (Figure 4A). If the increase in mortality with time is linear, this results in a half-life of approximately three years for a seed in the soil, on average.

Germination of seeds in the soil seed bank did not differ among regions or populations (*P*>0.10 in both cases), but differed significantly among retrieval times (*P*<0.0001). Seed germination values were very high in the first autumn, six months after burial (mean±SE percent germination across populations  = 81.9±2.4%), sharply decreased in spring after one year (14.4±5.7%), and peaked again but to a lesser extent in the second autumn (59.7±7.8%). However, the decrease of seed germination in spring was more pronounced and less variable in montane (0.7±0.2%) than in coastal populations (26.5±13.0%; Figure 4B). In agreement with this, when differences for seed germination between regions were tested at each retrieval time separately by one-way ANOVAs, the only significant result was found in spring (*P* = 0.005).

### Demographic traits: aboveground individuals

**Figure 5:**
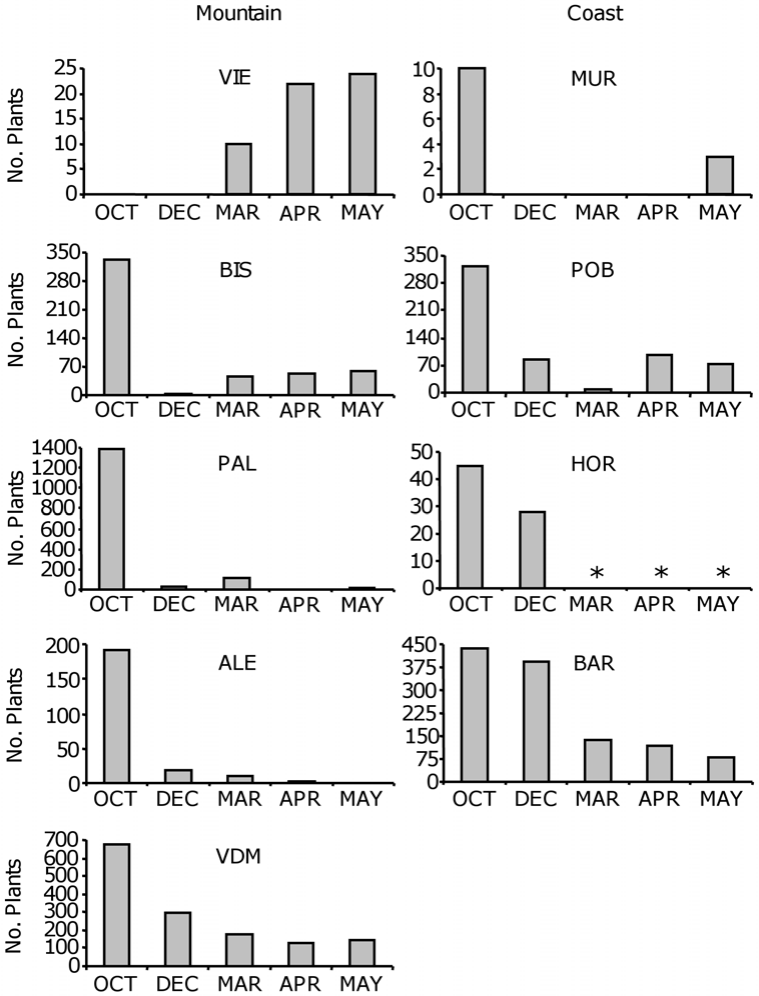
Total number of plants per population over time. The different panels show the number of plants per population and survey (October 2007, December 2007, March 2008, April 2008, and May 2008) for montane and coastal *A. thaliana* populations. Asterisks indicate that data were not available at HOR because the site was destroyed in late winter. Populations are ranked according to their altitude within each region.

The total number of plants across populations ranged from a minimum of 432 (April 2008) to a maximum of 3695 (October 2007), totaling 6062 plants recorded and measured during the entire demographic study. Population size was significantly different throughout the year (*P*<0.0001) and among populations (*P*<0.0001), the interaction between population and time being also significant (*P*<0.0001). Overall, the dynamics of populations based on periodic surveys indicated that population size peaked in autumn, sharply decreased in winter and in some populations slightly increased again in spring (Figure 5). We detected new plants recruited in March, April and/or May censuses in all populations, except in ALE that gradually lost all individuals throughout the year (Figure 5). The final number of reproductive plants in spring was in some cases a mixture of autumn and spring germinated plants (e.g.: VDM, BAR) or almost exclusively composed of spring germinated plants (e.g.: VIE, BIS, POB, MUR).

*Arabidopsis thaliana* populations experienced a pronounced reduction in size between autumn and winter after the recruitment peak in autumn (except VIE; Figure 5). The change in plant density *ΔN* between autumn and winter differed significantly between regions (*P* = 0.001; Table 2) but did not differ among populations within regions (*P* = 0.76). The number of plants per plot in October significantly affected *ΔN* between autumn and winter (*P*<0.0001): plots with more plants had higher reductions in population size in both montane (*r* = −0.34, *P* = 0.012) and coastal (*r* = −0.51, *P*<0.0001) populations. Based on these correlation coefficients, the effect of plant density in October on change in population size was stronger in coastal than in montane populations. Population size reductions in montane populations (mean±SE percent reduction  = 82.9±10.6%) nearly doubled those of coastal populations (44.0±27.7%). The *ΔN* values mainly included mortality but also some recruitment between the October and December surveys. A total of 18.2% of plots (18 of 99 plots with plants in October and/or December) had positive *ΔN* indicating that recruitment was more important than mortality in these plots. A total of 89% of plots (16 of 18) with positive *ΔN* were found in coastal populations (BAR, HOR and POB) whereas the remaining 11% (2 of 18) were found in montane populations (VDM and PAL). In winter, the number of leaves per rosette did not differ between regions (P = 0.21) but showed significant differences among populations within regions (*P*<0.0001; Table 2). The smallest and largest plants recorded across populations bore two and 14 leaves per plant, respectively.

**Table 2:** Sample size and mean (± SE) values for change in plant density and mean number of leaves of *A. thaliana*.

Region	Population	N	Change in plant density	N	Mean number of leaves
**Mountain**	**ALE**	16	−1.80 (0.24)	19	5.74 (0.46) a
	**BIS**	7	−2.19 (0.41)	–	–
	**PAL**	13	−2.24 (0.44)	27	2.00 (0.00) b
	**VDM**	18	−1.58 (0.29)	296	4.76 (0.07) ab
	**VIE**	–	–	–	–
**Coast**	**BAR**	17	−0.20 (0.25)	393	5.65 (0.10) a
	**HOR**	14	−0.19 (0.26)	28	5.89 (0.39) a
	**MUR**	–	–	–	–
	**POB**	14	−0.83 (0.57)	86	4.80 (0.17) ab
**Mountain**		4	**−1.95 (0.16)**	3	4.17 (1.12)
**Coast**		3	**−0.41 (0.21)**	3	5.45 (0.33)

Means with different letters differ significantly from one another. Average (± SE) values for montane and coastal populations are indicated below. Average values in bold face differ significantly from one another (*P*<0.05).

We did not observe reproductive plants in any population of study until March. In early March, the coastal populations BAR and HOR had many plants with flowering buds (65% in BAR, data from the HOR transect not available but observed in off-transect plants) whereas some others plants already bore flowers and fruits (35% in BAR). The rest of populations only had vegetative plants at this survey time. In early April, 62% of plants from the coastal BAR ceased to flower and bore fruits only. All plants from the other two coastal populations POB and MUR were in the middle of the reproductive season possessing buds, flowers and fruits. In early April, the montane VIE and BIS populations showed 76% and 15% of reproductive plants, respectively, mostly with buds but a few with the first open flower. The other montane PAL and ALE populations had only vegetative plants whereas 96% of plants from VDM were in the middle of the reproductive season. All coastal populations finished fruiting within the first half of May whereas montane populations finished within the second half of May and the first week of June. By the end of June, practically all plants from all populations had shed seed.

None of the fecundity parameters significantly differed between regions (all P>0.19; Table 3) but showed differences among populations within regions (all P<0.0001). Among populations, the mean number of filled fruits per plant ranged 1.4–14.4, the percentage of aborted flowers 0.0–9.3%, the percentage of depredated fruits 0.0–4.6%, the mean number of seeds per fruit 29.0–43.4, and the mean seed weight 2.0–2.9×10^−5^ g (Table 3). Overall, maternal plant size was positively correlated with mean number of seeds per fruit (*r* = 0.70, P<0.0001) as well as with mean seed weight (*r* = 0.39, P<0.0001).

**Table 3:** Sample size and mean (± SE) values for reproductive parameters of *A. thaliana*.

Region	Population	N	Filled fruits	N	Aborted flowers (%)	N	Depredated fruits (%)	N	Seeds per fruit	N	Seed weight (×10^−5^ g)
**Mountain**	**ALE**	–	–	–	–	–	–	14	40.5 (3.32) a	13	2.01 (0.11) b
	**BIS**	53	9.19 (1.02) a	53	9.32 (2.75) a	53	4.64 (1.95) a	15	43.4 (1.63) a	15	2.93 (0.14) a
	**PAL**	–	–	–	–	–	–	15	33.4 (2.26) ab	15	2.61 (0.16) ab
	**VDM**	145	2.46 (0.16) c	145	0.48 (0.37) c	145	0.37 (0.37) ab	–	–	–	–
	**VIE**	31	9.35 (1.52) a	31	5.19 (2.19) b	31	1.23 (0.73) ab	19	36.5 (1.14) ab	19	2.90 (0.21) ab
**Coast**	**BAR**	79	9.53 (1.91) b	79	0.73 (0.55) c	79	0.49 (0.35) ab	14	32.9 (1.99) ab	14	2.08 (0.10) b
	**HOR**	153	14.35 (1.35) a	153	1.58 (0.50) c	153	3.06 (0.82) ab	14	41.2 (2.25) a	14	2.42 (0.14) ab
	**MUR**	–	–	–	–	–	–	16	29.0 (2.11) b	15	2.33 (0.11) ab
	**POB**	75	1.41 (0.08) d	75	0.00 (0.00) c	75	0.00 (0.00) c	15	34.7 (3.00) ab	15	2.40 (0.14) ab
**Mountain**		3	7.00 (2.27)	3	5.00 (2.55)	3	2.08 (1.30)	4	38.44 (2.22)	4	2.61 (0.25)
**Coast**		3	8.43 (3.78)	3	0.77 (0.46)	3	1.18 (0.95)	4	34.44 (2.54)	4	2.31 (0.90)

Means with different letters differ significantly from one another. Average (± SE) values for montane and coastal populations are indicated below. Average values in bold face differ significantly from one another (*P*<0.05).

### Genetic diversity, differentiation, and structure

Coastal populations showed significantly higher genetic diversity than montane populations (*P*<0.019 for all parameters; Table 4). In comparison with montane populations, coastal populations doubled the proportion of polymorphic loci, doubled the number of multilocus genotypes, and almost tripled the gene diversity estimates (Table 4). Coastal populations had significantly higher mean number of alleles, higher allelic richness and higher private allelic richness than montane populations (Table 4). No identical multilocus genotype was found among populations. Mean differences among multilocus genotypes ranged 12.1–20.7% of the markers among coastal populations and 9.0–14.1% among montane populations. On average, the proportion of markers that occurred as singletons within a population was 15.8±3.9% and 18.2±11.7% for coastal and montane populations, respectively.

**Table 4:** Sample size and mean (± SD) values for genetic diversity parameters of *A. thaliana*.

Region	Population	N	Polymorphic loci	Number of alleles	Allelic richness	Private allelic richness	Gene diversity	Multilocus genotypes
**Mountain**	**ALE**	20	39.2	1.39 (0.49)	1.32 (0.43)	0.001 (0.008)	0.14 (0.20)	9
	**BIS**	13	0.0	1.00 (0.00)	1.00 (0.00)	0.000 (0.000)	0.00 (0.00)	1
	**PAL**	18	33.3	1.34 (0.48)	1.20 (0.31)	0.005 (0.040)	0.07 (0.12)	9
	**VDM**	19	18.0	1.17 (0.38)	1.15 (0.35)	0.008 (0.055)	0.07 (0.16)	4
	**VIE**	20	20.3	1.21 (0.41)	1.18 (0.36)	0.006 (0.045)	0.08 (0.15)	4
**Coast**	**BAR**	19	53.2	1.55 (0.50)	1.43 (0.43)	0.028 (0.134)	0.19 (0.21)	13
	**COC**	20	78.5	1.78 (0.42)	1.61 (0.38)	0.070 (0.198)	0.26 (0.19)	17
	**HOR**	20	55.7	1.57 (0.50)	1.44 (0.43)	0.041 (0.140)	0.19 (0.20)	11
	**MUR**	20	41.8	1.43 (0.50)	1.36 (0.44)	0.020 (0.123)	0.16 (0.21)	10
	**POB**	19	53.2	1.55 (0.50)	1.46 (0.44)	0.020 (0.115)	0.20 (0.21)	8
**Mountain**		5	**22.2 (6.8)**	**1.22 (0.07)**	**1.17 (0.05)**	**0.004 (0.002)**	**0.07 (0.02)**	**5.4 (1.6)**
**Coast**		5	**56.5 (6.0)**	**1.58 (0.06)**	**1.46 (0.04)**	**0.036 (0.009)**	**0.20 (0.02)**	**11.8 (1.5)**

Average (± SE) values for montane and coastal populations are indicated below. Average values in bold face differ significantly from one another (*P*<0.05).

AMOVA analysis showed significant genetic differentiation between coastal and montane regions corresponding to 7.4% of the total genetic variation (*P* = 0.008). The proportion of genetic variation among populations within regions was 30.8% (*P*<0.0001) while 61.8% (*P*<0.0001) of the variation was present among individuals within populations. All population-pair *F_ST_* values were significant (*P*<0.0001) and ranged 0.152–0.356 among coastal populations (mean population-pair *F_ST_*  = 0.266) and 0.263–0.677 among montane populations (mean population-pair *F_ST_*  = 0.494). Mantel tests did not detect significant correlation between genetic and geographic distances when analyzing all populations (*P* = 0.23), or in analyses of coastal and montane populations separately (*P* = 0.61 and 0.22 for coastal and montane populations, respectively).

## Discussion

Our demographic approach aims at disentangling the interplay of variation in environmental conditions, demographic patterns and genetic composition of *Arabidopsis thaliana* populations to provide insights into the differential functioning of populations across the species' native environmental range. This knowledge on the population biology of *A. thaliana in situ* is capital for understanding the environments in which life-history traits have evolved and the consequences for population dynamics and genetic composition, major components of evolutionary change. This study represents the first stage of that disentangling: a description of the system attributes that opens the door to more detailed mechanistic and causal studies.

As far as the environmental variation is concerned, coastal and montane *A. thaliana* populations clearly differ in their climatic patterns. It is well accepted that climate has major effects on the geographical range of many plant species [44], [45], and in the case of *A. thaliana* it has been suggested that temperature and precipitation regimes may be the most important factors in determining the species-wide environmental range [46]. Our study suggests again, this time on a regional scale, the effects that variation in temperature and precipitation may have on variation in demographic and genetic characteristics of *A. thaliana* populations along a wide environmental range from low-elevation Mediterranean to high-elevation Pyrenean sites. Vegetation and soil differences between coastal and montane populations are less pronounced and could be accounted for by the different disturbances at the study sites. Disturbances, such as grazing in montane semi-natural grasslands or track maintenance in some coastal populations likely prevent the closure of gaps, create germination safe sites, bring buried seeds to the surface and can contribute to maintaining *A. thaliana* populations by keeping low levels of interspecific competition.

From a demographic point of view, some demographic traits exhibit significant variation between coastal and montane regions. One of these traits is the seasonal change in dormancy of seeds buried in the soil seed bank. Our experiment indicates that seeds buried in the soil acquire strong secondary dormancy in late spring and regain the ability to germinate every autumn. Secondary dormancy prevents late germination of seeds and can be lost and reacquired repeatedly as seasons change until the required germination conditions become available [47]. Our results indicate that secondary dormancy tends to be stronger for montane than for coastal populations. In agreement with [30], who pointed out that cold winter temperatures in the absence of light can induce seed dormancy in *A. thaliana*, we suggest that colder winter temperatures in montane areas probably contribute to the stronger secondary dormancy and lower spring germination of seeds in the soil seed bank in montane populations.

A more general pattern that emerges from our soil seed bank experiment and that improves our understanding on the ecology of *A. thaliana* includes a high persistence of seeds buried in the soil but also a high germination fraction. This latter trait is normally associated with low temporal variation in reproductive success and therefore lower risk when attempting to reproduce [48], [49], suggesting that long-term persistence of *A. thaliana* populations may be to some extent conditioned by yearly seed production that replenishes the seed bank. In addition, the seed bank can be heterogeneous, i.e.: mosaic seed banks with areas of high and low seed persistence, seed mortality and/or emergence [50], as shown by the differential pattern of seed mortality at the MUR population, which might be attributed to particular environmental conditions at this site, such as shallow soil on a SW facing slope.

The most important demographic difference between coastal and montane *A. thaliana* populations has to do with population shrinkage in winter after the recruitment peak in autumn observed in almost every population at both regions. High seedling/juvenile mortality due to abiotic and/or biotic factors in different seasons of the year is a well-known result in plant population biology [51]–[53]. Reductions in population size between autumn and winter in montane populations almost double those recorded in coastal populations. We suggest that colder winters in montane sites, with mean minimum temperatures below zero from November to March, account for higher plant mortality in montane than in coastal populations. Interestingly, the effects of plant density on plant mortality are higher in coastal than in montane sites, suggesting that coastal populations could show stronger intraspecific density-dependent mortality. Hence, we hypothesize that higher plant density due to lower abiotic mortality could increase intraspecific density-dependent mortality in coastal populations.

Despite maximum germination during autumn, we observed new plants after winter, in March, April and/or early May in almost all populations, which indicates that *A. thaliana* populations are admixtures of several cohorts. This raises the question of which is the life habit of natural populations of *A. thaliana*. Qualitatively, most studied populations in this work should be considered as winter annuals because they began germination in autumn, in agreement with other observations [18], [30]. However, our data clearly show that several populations in the year of study depend on late winter and early spring germination to recruit plants that finish the reproductive cycle. Therefore, we support the view that *A. thaliana* populations behave as quantitative variable admixtures of autumn to spring germinating plants. Our results suggest that variation for germination timing is a major determinant contributing to the dynamics of *A. thaliana* despite the previous observation that fitness (in terms of survivorship and reproductive output) can be lower for spring-germinated than for autumn-germinated plants [54].

Finally, coastal and montane populations also differ in flowering time, in particular coastal BAR and HOR populations flower earlier than the rest of populations. In *A. thaliana* it is well documented that long days, high ambient temperature and vernalization (i.e.: exposure to low temperatures in winter) promote flowering [55], [56]. Given the narrow latitudinal distances between coastal and montane populations and the mild winters in coastal sites, it can be hypothesized that high temperatures in late winter contribute to accelerated flowering in these coastal populations. Despite this pattern of variation in flowering time, we do not find regional differences either for plant size in winter or for any of the reproductive traits. Therefore, the significant variation observed among populations suggests that population-specific environmental factors (e.g.: microhabitat variation) could be more important than regional factors in determining vegetative and reproductive traits in *A. thaliana*. Such microhabitat variation and its effects on plant traits has to be analyzed and understood because plant size, given by the total number of fruits, is a good predictor of the number of seeds per fruit [57, this study], which is a principal fitness component in *A. thaliana*.

Genetically, montane populations are differentiated from coastal populations in putatively neutral SNP markers, and populations within regions are even more strongly differentiated, especially in the montane region. The genetic differentiation found in this study is supported by the fact that no shared multilocus genotypes were found among *A. thaliana* populations indicating strong population isolation, in accordance with other studies on genetic diversity of *A. thaliana* natural populations [15], [58]–[60]. Hence, the selfing nature of the species and the physical isolation of populations may easily lead to rapid genetic differentiation in *A. thaliana*.

A previous study on the geographic structure of genetic variation of *A. thaliana* populations in the Iberian Peninsula showed strong isolation-by-distance patterns, which identified major geographical barriers [15]. Populations in NE Spain belonged to a single genetic group and showed the strongest differentiation from populations of other geographical regions suggesting a different history of isolation in NE Spain [15]. Hence, we speculate that *A. thaliana* populations in NE Spain, such as the populations of this study, share a common history perhaps driven from the last Pleistocene glaciations. The absence of isolation-by-distance in our set of study populations in combination with the observed population differentiation reinforces the view of strong population isolation and subsequent differentiation due to dispersal limitation. Such processes might differ between coastal and montane populations because on average genetic differentiation among coastal populations is lower than that among montane populations. In general, *A. thaliana* is more abundant is coastal than in mountain sites (FX Picó, *pers. obs.*), which could lead to weaker isolation and lower genetic differentiation in coastal areas.

Montane populations are also genetically less diverse than coastal populations, since all genetic diversity parameters are higher in coastal populations. Numerous demographic processes shape population patterns of genetic diversity by changing effective population size [61], and population size is a well-documented predictor of the amount of genetic variation [62], [63]. The probability of losing genotypes due to elevated mortality after the recruitment peak in autumn is higher in montane than in coastal populations. Thus the genetic composition of montane populations depends more strictly on spring germination and recruitment, which are less intense than those in autumn. Hence, our hypothesis is that climate-mediated variation in key demographic traits, such as juvenile mortality and recruitment, can account for the differential amount of neutral genetic diversity between coastal and montane *A. thaliana* populations.

### Conclusions

We have shown that demographic performance and genetic composition and differentiation of *A. thaliana* populations, albeit low, significantly differ between coastal and montane regions. We have found that between-region differences in climatic patterns are consistent with the differential demographic performance of coastal and montane populations. Furthermore, such demographic differences could partly account for the differential genetic patterns of variation between regions. It is of importance to increase our understanding on the population biology of *A. thaliana* by incorporating the temporal and spatial variation (i.e.: variation among patches within a population) in demographic and genetic parameters and analyzing the consequences of that variation for the long-term dynamics of local populations. Furthermore, assessment of the extent of local adaptation of *A. thaliana* to coastal and montane environments is also needed to identify the traits that can become targets of an evolutionary and ecological functional genomics approach.
